# Cathepsin gene expression in abdominal subcutaneous adipose tissue of obese/overweight humans

**DOI:** 10.1080/21623945.2020.1775035

**Published:** 2020-06-02

**Authors:** Qing Xu, Edwin C. M. Mariman, Gijs H. Goossens, Ellen E. Blaak, Johan W. E. Jocken

**Affiliations:** Department of Human Biology, NUTRIM School of Nutrition and Translational Research in Metabolism, Maastricht University Medical Centre^+^, Maastricht, The Netherlands

**Keywords:** Adipose tissue, autophagy, lysosome, obesity, glucometabolic status

## Abstract

Cathepsin L1 (CTSL1) and B (CTSB) are lysosomal proteases, of which the expression and activity are impaired in adipose tissue (AT) of obese rodents, indicating AT lysosomal dysfunction. Here we assess the relation between abdominal subcutaneous AT (SCAT) CTSL1 and CTSB *gene* expression (qRT-PCR), body composition and tissue-specific insulin resistance in 77 overweight/obese (BMI: 225.6–38.6 kg/m^2^) well phenotyped men and women (61 M/16 F). A two-step hyperinsulinemic-euglycemic clamp was performed to assess AT, hepatic and skeletal muscle insulin sensitivity. Our data show that reduced CTSB expression is associated with markers of insulin resistance (standardized β = −0.561, p < 0.001), independent of adiposity, while CTSL1 expression is only associated with markers of body composition. Our data suggest the presence of lysosomal dysfunction in SCAT of obese humans with an impaired glucose homoeostasis. However, this needs to be investigated in more detail in future mechanistic studies.

## Introduction

Impaired regulation of adipose tissue (AT) lipid metabolism may lead to excess lipid accumulation in ectopic tissues (e.g. skeletal muscle and liver), which contributes to the development of obesity-associated insulin resistance [1]. Of interest, impaired regulation of autophagy, a recycling system to maintain intracellular homoeostasis, has often been observed in AT of obese humans and rodents [2,3]. Recently, we showed that increased expression of autophagic genes (i.e. ATG 5–12 and 7) in human subcutaneous AT, was associated with an impaired glucometabolic status [4]. Lysosomal degradation of the autophagic cargo is the final stage of autophagy. Lysosomes contain over 50 functional enzymes (including glucosidases, proteases, sulfatases and others) for intracellular degradation of cellular components [5], which play an essential role in maintaining the autophagic clearance in adipose tissue [6]. Therefore, lysosomal dysfunction might play a key role in obesity-related metabolic disorders.

Cathepsins are a group of lysosomal proteases responsible for maintaining intracellular homoeostasis [7]. However, Cathepsin L1 (CTSL1) and Cathepsin B (CTSB) are the most abundant lysosomal proteases and participate directly in the execution of autophagy [8]. In addition, they have recently been implicated in lysosomal dysfunction and early pathologies of obese murine adipose tissue [7,9]. Recently, it was found that enhanced activation of CTSB protein and a concomitant decreased CTSL1 activation in AT is a marker of AT lysosome dysfunction in obese rodents, resulting in an attenuated lysosomal clearance and autophagosome accumulation [9]. Of interest, increased CTSB and CTSL1 mRNA expression was observed in the white adipose tissue of genetic (ob/ob) and high fat diet-induced obese mice, possibly contributing to obesity-associated adipose tissue dysfunction [9]. Therefore, CTSB and CTSL were of primary interest for our human explorative study in adipose tissue of obese metabolically compromised individuals. However, human evidence for lysosomal dysfunction within the obese adipose tissue is still scarce [9]. In this study, we performed a cross-sectional analysis in subcutaneous adipose tissue samples derived from 77 well phenotyped overweight/obese men and women of whom tissue-specific insulin sensitivity was determined using the gold standard hyperinsulinemic-euglycemic clamp with [6,6–^2^H_2_]-glucose infusion [10]. CTSL1 and CTSB gene expression was measured using quantitative real time PCR (18 S was used as a housekeeping gene and data were calculated using delta CT method [11]) and related to detailed measures of glucose homoeostasis and adiposity.

## Materials and methods

### Study population

Subcutaneous AT (SCAT) samples were derived from two independent cohorts [12,13]. In total 77 low active (<3 h organized sports activities per week), weight-stable (<2 kg body weight change 3 months prior to inclusion) overweight/obese Caucasian individuals (BMI 25–39 kg/m^2^) without type 2 diabetes were included. Cohort 1 consists of 50 men, age between 35 and 69 years with impaired fasting glucose (fasting glucose ≥6.1 mmol/L) and/or impaired glucose tolerance (2 h plasma glucose during a 75 g glucose tolerance test 7.8–11 mmol/L) and HOMA-IR>2.2. Cohort 2 consists of 27 subjects, men (n = 11) and women (n = 16) age between 21 and 50 years with normal glucose metabolism (fasting <6,1 mmol/L and 2 h < 7.8 mmol/L). Our well-phenotyped cohorts included 19 overweight men and 8 overweight women, and 42 obese men and 8 obese women. Exclusion criteria were smoking, cardiovascular disease, cancer, lung disease, intentions to lose weight, alcohol/drug abuse, use of antioxidants, and use of medication known to affect glucose metabolism or inflammation. The local Medical Ethical Committee of Maastricht University Medical Centre approved the study protocols (ClinicalTrials.gov NCT02241421 and NCT02381145) and all participants gave a written informed consent in advance.

### In vivo phenotyping and biochemical analysis

Anthropometric phenotyping was performed as described before [10,13]. Briefly, body weight was measured accurate to 0.1 kg on an electronic scale (Seca model 861, Hamburg, Germany) and height was measured accurate to 0.01 m. Hip and waist circumferences were measured over the greater trochanters and above the belly button below the rib cage, respectively. Waist/hip ratio (WHR) was then calculated. Blood was collected into pre-chilled tubes and centrifuged at 1,000x g, and plasma was snap-frozen and stored at −80°C until analyses. Plasma glucose and FFA were determined using commercially available colorimetric assays on a Cobas Fara auto-analyser (Roche, Switzerland). Plasma insulin was measured with a double antibody radioimmunoassay (Millipore). Fasting insulin sensitivity was assessed by calculating the HOMA-IR index using the formula described by Matthews et al [14].

### Hyperinsulinemic-euglycemic clamp

A two-step hyperinsulinemic-euglycemic clamp combined with a [6,6–^2^H_2_]-glucose tracer (Cambridge Isotope Laboratories) was performed to analyse the insulin-mediated suppression of free fatty acids (FFA suppression, representing AT insulin sensitivity), insulin-stimulated rate of disappearance of glucose (RdSS, representing peripheral/muscle insulin sensitivity), and insulin-mediated suppression of endogenous glucose production (%EGP suppression, representing hepatic insulin sensitivity). After a bolus-injection (2.4 mg/kg) [6,6–^2^H_2_]-glucose, tracer-infusion was started at 0.04 mg/kg/min, which was continued throughout the measurement. After 2 hours, low-dose insulin was infused at 10 mU/m^2^/min for 2 hours [15], followed by high-dose insulin at 40 mU/m^2^/min for 2 hours [16]. Blood samples were taken from a superficial dorsal hand vein, which was arterialized using a hot-box (50°C). By variable co-infusion of a 17.5% glucose solution, enriched by 1.1% tracer, plasma glucose concentrations were maintained at 5.0 mmol/l. For calculation of steady-state kinetics, three additional blood samples were taken every 10 min in the last 30 min of each step (0, 10, and 40 mU/m^2^/min insulin). The Rd was calculated during the 0- and 40-mU/m^2^/min insulin infusion, whereas calculations for insulin-mediated suppression of EGP and FFAs were performed during 0-and 10-mU/m^2^/min insulin infusion, as relative percentage of suppression during 10 compared with 0 mU/m^2^/min [10].

### Adipose tissue biopsy

Abdominal subcutaneous AT (SCAT) biopsies were collected 6 to 8 cm lateral from the umbilicus under local anaesthesia (2% lidocaine) by needle biopsy after an overnight fast. After immediate washing with saline, biopsy material was snap-frozen in liquid nitrogen and stored at −80°C until mRNA analysis [13].

### Adipose tissue mRNA analysis

Total RNA was extracted from tissue samples using TRIzol® Reagent (Ambion/Life Technologies; 15,596–026). Reverse transcription of 300 ng of total RNA was performed using the iScript cDNA synthesis kit (Bio-Rad; 170–8891). SYBR-Green-based real-time PCRs were performed using an *iCycler* iQ Real Time PCR detection system (Bio-Rad). Reactions were performed in a total volume of 25 μl containing 5.5 μl cDNA, 12.5 μl iQ SYBR green supermix (BIO-RAD; 1,708,882) and gene-specific primers for CTSL1 (Biolegio, Forward Primer AAGTGGAAGGCGATGCACAA, Reverse Primer AAAGCCATTCATCACCTGCC) and CTSB (Biolegio, Forward Primer TCGGATGAGCTGGTCAACTA, Reverse Primer AGCTTCAGGTCCTCGGTAAA). 18 S was used as a housekeeping gene and data were calculated using delta CT method [17].

### Statistical analysis

All variables were checked for normal distribution and variables with a skewed distribution were ln-transformed. Firstly, the Pearson’s correlation between CTSB and CTSL1 mRNA expression were tested. Next, the associations of clinical parameters and gene expression were tested by Pearson’s correlation. Secondly, univariate regression was performed with CTSB or CTSL1 gene expression as dependent variables and age, sex, BMI and WHR as independent variables (model 1). After that, to study the impact of CTSB or CTSL1 on glucometabolic status and insulin resistance, univariate regression analysis was performed with fasting insulin, fasting glucose, HOMA-IR or HbA1 C as dependent variables, and RNA expression of CTSB or CTSL1 as the independent factor adjusted by age, sex, BMI and WHR. Finally, to study the impact of CTSB or CTSL1 on tissue-specific insulin sensitivity, univariate regression analysis was performed with FFA suppression, EGP and Rd entered separately as dependent variables, and RNA expression of CTSB or CTSL1 as the independent factor adjusted by age, sex, BMI and WHR. Calculations were performed with SPSS 23.0 for Windows (SPSS Inc., Chicago, IL, USA). All tests for statistical significance were two-tailed, and p < 0.05 was considered statistically significant.

## Results

### Study population

Clinical characteristics of the 77 overweight/obese participants are summarized in Table 1. Briefly, 16 females and 61 male overweight/obese participants were enrolled with an age range between 21 and 69 years, and broad range in BMI (25.6–38.6 kg/m^2^), fasting glucose (4.5–7.5 mM), fasting insulin (2.8–29.3 mU/L), and whole body insulin resistance (HOMA-IR 0.65–8.76) and tissue-specific insulin resistance (see Table 1).

**Table 1.** Participants’ characteristics

	MEAN±SEM	RANGE
Male/female	61(42 obese)/16(8 obese)	
Age (years)	52 ± 2	21–69
Weight (kg)	94.8 ± 1.4	69.4–122.0
Height (m)	1.75 ± 0.01	1.52–1.92
BMI (kg/m^2^)	30.9 ± 0.3	25.6–38.6
Waist (cm)	104 ± 1	77–126
Hip (cm)	106 ± 1	89–125
WHR	0.98 ± 0.01	0.70–1.22
Fasting glucose (mM)	5.7 ± 0.1	4.5–7.5
Fasting insulin (mU/L)	13.9 ± 0.7	2.8–29.3
2 h glucose (mM)	6.7 ± 0.2	3.4–11.2
HOMA-IR	3.6 ± 0.2	0.7–8.8
HbA1 C (%)	5.4 ± 0.1	4.7–6.7
Fasting FFA (μM)	615 ± 16	399–960
FFA suppression (%)	52.1 ± 2.3	6.1–90.1
EGP suppression (%)	48.5 ± 2.2	5.2–87.2
Rd (μmol/kg/min)	26.3 ± 1.2	9.8–54.0

Data are mean ± SD [Range: min-max]; BMI: body mass index, WHR: waist-hip ratio, HOMA-IR: homoeostatic model assessment for insulin resistance, HbA1 C: haemoglobin A1 C, FFA: free fatty acids, EGP: endogenous glucose production, Rd: rate of disappearance.

### CTSB and CTSL1 expression, body composition and tissue-specific insulin sensitivity

A significant positive correlation was observed between CTSB and CTSL1 mRNA expression in human SCAT (r = 0.419, p < 0.001). As shown in Table 2, CTSB mRNA expression did not correlate with markers of body composition (including BMI and WHR), but correlated negatively with age (r = −0.261, p = 0.024), fasting glucose (r = −0.299, p = 0.009), fasting insulin (r = −0.343, p = 0.003), HOMA-IR (r = −0.378, p = 0.001) and fasting FFA (r = −0.271, p = 0.036). The negative relationship between CTSB expression and fasting glucose (standardized β = −0.345, p = 0.034), insulin (standardized β = −0.505, p < 0.001), and HOMA-IR (standardized β = −0.561, p < 0.001) remained significant after adjustment for age, sex, BMI and WHR (Table 4). However, no associations were observed between AT, skeletal muscle and hepatic insulin sensitivity and SCAT CTSB expression (Table 5).

**Table 2.** Pearson’s correlation coefficients for CTSB, CTSL1 gene expression and clinical characteristics

	Ln CTSB	Ln CTSL1
Age (years)	−0.261*	0.352**
BMI (kg/m^2^)	0.111	0.363**
WHR	−0.076	0.521***
Fasting glucose (mM)	−0.299**	0.230*
Fasting insulin (mU/L)	−0.343**	0.178
2 h glucose (mM)	−0.047	0.221
HOMA-IR	−0.378**	0.183
HbA1 C (%)	−0.033	0.250*
Fasting FFA (μM)	−0.271*	0.118
FFA suppression (%)	−0.005	−0.372**
EGP suppression (%)	0.020	−0.235*
Rd (μmol/kg/min)	0.044	−0.341**

*p < 0.05, **p < 0.01, ***p < 0.001,

**Table 3.** Relationship between CTSB, CTSL1 gene expression in SCAT and adiposity

	CTSB gene expression			CTSL gene expression		
Dependent Variables (n = 77)	standardized β [95%CI]	p value	Adj. R^2^	standardized β [95%CI]	p value	Adj. R^2^
BMI	0.110 [−0.119, 0.339]	0.345	0.012	0.369 [0.149, 0.588]	0.001**	0.131
Model 1						
BMI	0.104 [−0.159, 0.368]	0.433	0.009	0.196 [−0.039, 0.430]	0.101	0.040
Age	−0.430 [−0.814, −0.046]	0.029*	0.070	−0.080 [−0.414, 0.255]	0.635	0.003
Sex						
Men	0.128 [−0.900, 1.156]	0.805	0.001	0.404 [−0.499, 1.307]	0.375	0.012
Women	0			0		
WHR	0.170 [−0.409, 0.750]	0.560	0.005	0.378 [−0.131, 0.887]	0.143	0.032

All values entered in the model after z score standardization. *p < 0.05, **p < 0.01, ***p < 0.001

**Table 4.** Relationship between CTSB, CTSL1 gene expression in SCAT and circulating glucose and insulin concentrations

	Fasting glucose			Fasting insulin			HOMA-IR			HbA1 C		
standardized β [95%CI]	p value	Adj. R^2^	standardized β [95%CI]	p value	Adj. R^2^	standardized β [95%CI]	p value	Adj. R^2^	standardized β [95%CI]	p value	Adj. R^2^	
CTSB gene expression	−0.196[−0.376, −0.016]	0.034	0.068	−0.389[−0.584, −0.194]	0.000	0.196	−0.384[−0.566, −0.203]	0.000	0.216	0.057[−0.151, 0.265]	0.585	0.005
CTSL1 gene expression	−0.071[−0.293, 0.152]	0.527	0.006	−0.102[−0.354, 0.150]	0.423	0.010	−0.122[−0.364, 0.120]	0.317	0.015	0.081[−0.168, 0.330]	0.517	0.007

All values entered in the model after z score standardization and adjusted for age, sex, BMI, WHR.

**Table 5.** Relationship between CTSB, CTSL1 gene expression in SCAT and tissue-specific insulin sensitivity

	FFA suppression			EGP suppression			RdSS		
Dependent Variables (n = 77)	standardized β [95%CI]	p value	Adj. R^2^	standardized β [95%CI]	p value	Adj. R^2^	standardized β [95%CI]	p value	Adj. R^2^
CTSB gene expression	0.029[−0.166,0.225]	0.765	0.001	0.054[−0.187,0.295]	0.656	0.003	−0.013[−0.213,0.188]	0.901	0.000
CTSL1 gene expression	−0.018[−0.227,0.191]	0.864	0.000	0.066[−0.198,0.331]	0.617	0.004	0.000[−0.226,0.226]	0.999	0.000

All values entered in the model after z score standardization and adjusted for age, sex, BMI, WHR.

In contrast, CTSL1 expression correlated positively with age (r = 0.352, p = 0.002), BMI (r = 0.363, p = 0.001), WHR (r = 0.521, p < 0.001), fasting glucose (r = 0.230, p = 0.046) and HbA1 C (r = 0.250, p = 0.031) (Table 2). Furthermore, CTSL1 expression correlated negatively with insulin-induced FFA suppression (r = −0.372, p = 0.001), EGP suppression (r = −0.235, p = 0.049) and Rd (r = −0.341, p = 0.003) (Table 2). However, after adjustment age, sex, BMI, WHR, the associations between CTSL1 SCAT expression and tissue-specific insulin sensitivity were no longer statistically significant (Tables 3–5).

## Discussion

In the present study, we examined the relationship between gene expression of the lysosomal proteases CTSB and CTSL1 in human SCAT, adiposity (i.e. BMI and WHR) and tissue-specific insulin sensitivity.in overweight/obese men and women. CTSB and CTSL1 mRNA expression in human SCAT were positively associated. Interestingly, a reduced SCAT CTSB mRNA expression was associated with increased whole-body insulin resistance (i.e. fasting glucose, insulin and HOMA-IR), independent of adiposity, while an increased SCAT CTSL1 mRNA expression was only associated with adiposity (i.e. BMI and WHR). However, both CTSB and CTSL1 expressions in the SCAT showed no significant association with tissue-specific insulin sensitivity following adjustment for age, sex, BMI, WHR. Together, our data indicate lysosomal dysfunction in the human adipose tissue of obese metabolically compromised humans.

The lysosomal proteases CTSB and CTSL1 are active as cysteine endo- and exopeptidase, which are abundantly expressed at the gene and protein level in human adipose tissue [8,18]. High expression of CTSB might contribute to increased basal lipolysis and a possible subsequent inflammatory response via reduce PLIN1 expression as shown in 3T3L-1 adipocytes [19]. Previously, observations in a relatively small study (n = 9) showed that CTSB gene expression is upregulated in SCAT of obese compared to lean 21–35 years-old men [20]. In contrast, the present data showed that CTSB expression in SCAT did not correlate with BMI or WHR in a large middle-aged population (age 52 ± 2 years).

It is well known that obesity is associated with insulin resistance of adipose tissue, the liver and skeletal muscle, reflected by whole-body insulin resistance [21]. However, the relation between SCAT lysosomal dysfunction and insulin resistance in obesity/overweight individuals has not been well studied. Autophagy is rapidly induced by nutrient deprivation (fasting) and evidence is accumulating that this fasting-induced autophagy is defective regulated in insulin sensitive tissues such as liver, muscle, and adipose, in the context of obesity, which underpins an unprecedented role of autophagy in the manifestation of obesity-induced metabolic derangement [22,23]. Here we show that CTSB mRNA expression in SCAT is negatively correlated with fasting glucose and insulin levels, while no associations were observed between SCAT CTSB mRNA expression and tissue-specific insulin sensitivity, as determined by a 2-step hyperinsulinemic euglycemic clamp. In contrast, in rodent models it has been reported that whole-body pharmacological and genetic inactivation of CTSB protected against the development of hepatic steatosis and whole-body insulin resistance [24]. We previously observed in a sub-study of this cohort that plasma Cathepsin D (CTSD) activity, but not systemic inflammation, is inversely related to hepatic insulin sensitivity, suggesting that plasma CTSD activity may be used as a non-invasive marker for hepatic insulin sensitivity in humans [25]. The observed fasting lysosomal dysfunction might compromise the ability of the cell to perform quality control on the mitochondrial matrix, since autophagy plays a pivotal role in the degradation of defective mitochondria. Similarly, autophagy also plays an indispensable role in the clearance of protein aggregates and redundant large protein platforms such as inflammasomes. Furthermore, autophagy might also play a key role in the metabolism of endotoxins, implicating the importance of autophagy in the pathogenesis of metabolic endotoxemia [26]. Together, these data argue for species-specificity concerning the action of cathepsins on the development of whole-body insulin resistance, which is important to recognize and warrants further investigation.

Finally, we observed that in abdominal SCAT of overweight/obese men and women, expression of CTSL1 was positively associated with adiposity). This finding is in line with a previous study in diet-induced and genetically obese mice (ob/ob mice), which were characterized by lower CTSL1 protein expression and activity in white AT (WAT) compared to lean control animals, while the mRNA expression of CTSL1 and pro-CTSL1 was increased in WAT of obese mice [9]. These data point towards an impaired lysosomal function in human obesity. However, this needs to be investigated in more detail in future research using functional measurements of SCAT lysosomal activity.

Of interest, we observed a strong positive correlation between CTSL1 and CTSB expression in human SCAT. However, pharmacological inhibition or genetic knockdown of CTSL1 induced a compensatory transcriptional upregulation and enzymatic activation of CTSB in murine 3T3 L1 adipocytes, which was accompanied by increased autophagosome accumulation, possibly reflecting lysosomal dysfunction [9]. In contrast to CTSL1, CTSB protein expression and its enzymatic activity were increased in WAT of HFD-induced obese mice [9]. This enhanced activation of CTSB promotes CTSL cleavage, resulting in further suppression of CTSL enzymatic activity, which leads to impaired autophagic clearance and autophagosome accumulation in WAT of obese mice [18]. Therefore, it will be of important to establish whether the observed transcriptional changes in human SCAT also translate into changes at the protein level and are accompanied by changes in CTSL1 and CTSB activity in obese/overweight men and women.

In future studies, it would be of interest to also investigate other cathepsin families in relation to human adipose tissue lysosomal dysfunction. In addition, it would be interesting to investigate cathepsin tissue expression, activity and plasma levels in lean as compared to overweight/obese humans, and include morbidly obese (BMI>35 kg/m2) individuals to investigate the effects of body weight and body composition per se. Moreover, future studies should also investigate the effect of lysosomal dysfunction on human adipocyte function and substrate metabolism using *in vitro* mechanistic approaches and to explore the road to modulate human adipose tissue lysosomal function via nutritional, pharmacological and lifestyle interventions aimed at improving metabolic health.

In summary, CTSL1 expression in human SCAT is positively associated with adiposity, while SCAT CTSB expression is inversely related to whole-body insulin resistance. Together, these data suggest that lysosomal dysfunction, reflected by increased CTSL1 and decreased CTSB expression, is present in SCAT of overweight/obese men and women, and may relate to impaired whole-body glucose homoeostasis. Nevertheless, our data are correlational in nature and further research is required to determine the causality of these associations.
